# Obstetrics and Gynæcology

**Published:** 1903-06

**Authors:** Walter C. Swayne


### OBSTETRICS AND GYNÆCOLOGY.

**Puerperal eclampsia** as a complication of labour is one of the most difficult subjects to deal with, on account of the great complexity of its pathology and of the numerous visceral lesions which accompany it. Our knowledge concerning it has advanced but little, in fact, is but little in advance of that of thirty years ago.

Bandler[1] shows that most of the theories which have been advanced as to its causation can be demonstrated to be incorrect, *e.g.*, the mechanical, uræmic, hydræmic, and anæmic theories, as to the causation of the affection, and the convulsions can be shown to have no solid basis. That irritation of the cerebral cortex has some influence in producing the convulsions is certain.

Blumreich and Zuntz[2] experimented on rabbits by applying kreatin to the motor areas of the cortex after removal of the bone and dura mater under anæsthesia. Convulsions occurred in all gravid animals, but in only 2 to 3 per cent. of the non-gravid, while it required three times as much kreatin in the non-gravid as in the gravid to produce convulsions. Injection of a solution of kreatin into the external carotid produced the same results.

"The central nervous system is therefore in a condition of extreme irritability during pregnancy."

Müller[3] observes that in eclampsia there is a rise in temperature, which increases with the frequency and severity of the convulsions, and sinks in the intervals between them. He also notes that epileptics are not specially liable to eclampsia.

He explains the symptoms on the theory of resorption fever, the poison being due to bacteria acting on uterine substances, causing their decomposition with the subsequent absorption of toxins into the circulation. The good results of intra-uterine death he attributes to diminution of intra-uterine pressure and consequent diminished absorption of toxins.

The frequency in primipara he attributes to the increase of intra-uterine pressure, due to the resistance of unstretched uterine muscle. He calls the kidney affection a toxic irregularity. Stroganoff[4] believes eclampsia to be an infectious disease, the contagion entering through the lungs. The incubation time is short—from five to twenty hours. It is especially liable to occur in lying-in hospitals. He considers it to be a constitutional disease and not a local one, and also independent of lesions of the kidney. He also believes that the contagion is caused by the medical attendant or nurse. He considers the important points to be the diminution of the convulsions, and relies on oxygen, morphia, chloral, and rapid delivery to effect this:

[1] *Am. J. Obst.*, 1903, xlvii. 433. [2] Quoted by Bandler, *loc. cit.*
[3] Quoted by Bandler, *loc. cit.* [4] Quoted by Bandler, *loc. cit.*

œdema of the lungs must be carefully guarded against. The pathological lesions of eclampsia are distinct and simulated by no other condition.

Schmorl goes so far as to say that cases occur in which death takes place without convulsions, *e.g.*, from coma and œdema of the lungs, and that these cases are truly eclamptic, because the pathological lesions present are those met with in eclampsia. On the other hand he excludes fatal convulsive cases in which the pathological lesions are absent. The lesions are—

(1) *Kidneys*: parenchymatous degenerations, glomerulitis and thrombi; (2) *Liver*: multiple hemorrhages and necrosis, and thrombi in the extra and intra-lobular branches of the portal vein; (3) *Heart*: hypertrophy of left ventricle, multiple hemorrhages and necrosis, and parenchymatous degenerations; (4) *Lung*: hyperæmia and œdema, thromboses and placental cell emboli; (5) *Brain*: punctate hemorrhages and areas of degeneration; (6) *Pancreas and adrenals*: hemorrhages and necroses.

Bile pigment is often present in the blood of eclamptics. Dienst[1] finds that similar lesions are present in both mother and fœtus. The fœtal kidneys scarcely secrete or perform any important function. *After birth*, however, the kidneys often prove to be unhealthy, albumin, hemalbumin and casts being found in the urine. He finds the amount of fibrin in the blood of eclamptic mothers and infants to be increased. He refers the cause to the fœtus, because the symptoms so often cease after delivery. This view is insufficient to explain those cases in which eclamptic seizures comes on after delivery.

Bandler[2] considers that the source of the substance which produces the lesions is to be found either in some structure of the patient herself, or in some element of the ovum. In the case of the one, the placenta and the fœtus itself; in the other, *the ovary*, since it is that organ whose excretion is inhibited by pregnancy, should be considered. He presents the following propositions—

(1) That the trophoblastic cells pass into the circulation; (2) that these constitute a placental secretion; (3) that the destruction of the trophoblasts indicates an antagonistic action of the maternal blood; (4) that we may grant that the fœtal and maternal elements both possess mutually destructive enzymes; (5) and that the production of eclampsia is a constitutional pathological malrelation between the maternal and fœtal enzymes.

Bandler allows that, even supposing that this proposition, namely, the antagonism of the fœtal and maternal enzymes is proved, we have yet to find definitely the productive element of eclampsia. He indicates that it is to be found in the absence of the regulating power of ovarian secretion.

Hence the eclampsia is the result (1) of a pathological

[1] Quoted by Bandler, *loc. cit.* [2] *Loc. cit.*

placental (excess), or ovarian (diminished) secretion, (2) a failure of antagonism between the two, and is in the truest sense an auto-intoxication. He also refers to the antagonistic action in many ways of ovarian and thyroid extract.

Partridge [1] sums up our knowledge of the pathology of eclampsia as follows: a toxæmia brought about by a changed metabolism of the liver and perhaps of other organs, and by a deficiency in excretion, and that *acetone* is one of the most active of the toxic products. He enlarges on the pathological *post-mortem* findings which have been already described by Bandler: as regards the pathology he shows no fresh evidence.

Aspell [2] investigates the question of constipation as an etiological factor in the production of eclampsia. He discusses the various etiological theories, and dismisses them on the ground that no single one will produce the eclamptic condition by itself; in particular, he calls attention to the important fact that kidney insufficiency alone is not a satisfactory explanation, and that convulsions have been known to occur in the fœtus of an eclamptic mother. He draws attention to the pulse and its tension as an indication of approaching danger, and cites a case in which with albuminuria higher pulse tension and headache appeared. He treated her by free catharsis, and even after twelve days purgation and a limited fluid diet she passed dark-coloured scybalous masses. (He examined the stools each day). He alludes to the well-known fact that eclamptic convulsions often occur after a heavy indigestible meal. He reports six cases, and in all of them marked constipation was present. He is of the opinion that the primary lesion is probably one of the liver.

A. Ascoli,[3] with a view to throwing new light upon the etiology of eclampsia, has conducted a series of interesting experiments. He immunised rabbits by injection or infusion of the placentæ of guinea-pigs, and also in another series by injection of the placentæ of their own series, and thus obtained two sera which he calls hetero-syncytiolysin (from guinea-pigs), and iso-syncytiolysin (from rabbits). He found that the syncytio-toxins resulting from the injection of placental tissue into the circulation can cause symptoms resembling those of eclampsia. This rather suggests that the idea which Bandler promulgates, that eclampsia depends in some way on the absorption of syncytial products, has a definite foundation, although the evidence does not appear convincing. The hetero-syncytiolysin produced by far the graver symptoms.

The idea that eclampsia is a renal disease pure and simple is now almost universally abandoned, and it is generally allowed that the condition is due to a toxæmia, the exact nature of

[1] *Am. J. Obst.*, 1903, xlvii. 313. [2] *Ibid.*, p. 483.
[3] *Centralbl. f. Gynäk.*, 1902, xxvi. 1321; abstract in *Am. J. Obst.*, 1903, xlvii. 558.

which is unknown, and that the failure of the various excreting organs, the liver, kidneys, lungs, and skin, to eliminate the toxin or toxins is the exciting cause of the affection.

The kidneys, after the failure of the liver to deal with a toxin circulating in the blood, become irritated and injured, so that they fail in their proper function, and, owing to defective secretion, urea is added to the other toxic elements circulating in the blood, while albumin appears in the urine. If the condition be not relieved eclampsia is one of the results which follow. This hypothesis, however, probable though it seems, is not sustained so far by conclusive experimental proof. The view that eclampsia is of bacterial, or rather of micro-organismic origin, has been again revived, it being attributed by Müller[1] to intra-uterine bacterial infection and analogous to septicæmic conditions. Albert[2] takes practically the same view, regarding the infection (?) as being due to the cessation of uterine drainage by the closure of the canal during pregnancy and raising of intra-uterine tension. Neither Müller nor Albert state what variety of micro-organism they have found in the cases which they examined, and their position must be looked upon as one very difficult to prove definitely. H. Oliphant Nicholson[3] believes that the principal symptoms of the eclamptic state are due to thyroid inadequacy. He shows that the normal enlargement of the thyroid gland present in pregnancy can be diminished or prevented by the administration of thyroid extract, and Lange has observed that albuminuria and eclampsia occurred in twenty out of twenty-five cases in which the usual thyroid hypertrophy did not occur. Iodothyrin favours metabolism and increases the excretion of urea, which, in eclampsia, is strikingly diminished, and, owing to a deficiency of iodothyrin, nitrogenous metabolism stops short of the formation of urea at a point where the products of metabolism are highly toxic.

The clinical features of eclampsia have such a marked resemblance to athyroidea that it may be regarded as a temporary form of the latter, and, while the athyroidea lasts, nitrogenous substances, owing to the inability of the liver and other organs to form urea, are turned into toxins, which injure the kidneys and still further diminish the urea excreted.

Iodothyrin is a diuretic and powerful vaso-dilator; hypothyroidism is always associated with contraction of the arteries, eclampsia almost invariably so, hence the toxæmia of pregnancy appears to be dependent upon the quantity and activity of the thyroid secretion, and the thyroid gland may be given a primary *rôle* in the causation of eclampsia.

Now, to sum up the results of these investigations. First, as usual, prevention is better than cure, and a recognition of the fact that the appearances of toxæmia and the pre-eclamptic stages are capable of diagnosis is most important. Many, if not

[1] Quoted by Fothergill, *Practitioner*, 1903, lxx. 219.
[2] *Ibid.*, p. 220. [3] Quoted by Fothergill, *loc. cit.*, p. 221.

most, of the minor disorders of pregnancy indicate toxæmia, as for instance vomiting, constipation, indigestion, headache, nervous irritabililty, disturbances of vision, and abnormal pigmentation, accompanied by constriction of the smaller blood-vessels and rise in blood-pressure, diminution in the quantity of urine passed and in the quantity of urea excreted. Albuminuria and œdema are more definite and more alarming signs, but an attempt should be made to make a diagnosis before their appearance. Too much stress cannot be laid on the importance of quantitative estimation of urea in every suspicious case. A very good rough rule is this, "that in any case in which the daily excretion of urea falls below 200 grains, the question of terminating pregnancy, if the condition does not improve under treatment, should be considered."

Nicholson would in every case, when the symptoms mentioned as belonging to the pre-eclamptic stage were present, at once commence the administration of thyroid-gland extract, grs. v. twice daily; and in his reported cases it was remarked that the symptoms of toxæmia recurred whenever the thyroid treatment was stopped, and disappeared again when it was recommenced. This is noteworthy as a piece of good experimental evidence. On this evidence alone one would feel inclined to make use of this treatment, of course as a supplement to purgation, fluid diet, and rest in any case in which the usual thyroid enlargement was absent. Aspell's cases simply furnish additional evidence as to the danger of constipation.

Bandler's opinions, taken with the experiments of Ascoli, would seem to show that the termination of pregnancy as a prophylactic is justifiable and necessary. We cannot regard the evidence produced by either as conclusive, although no doubt their researches are of importance, and, granting even a very limited amount of truth in the conclusions they draw, these certainly give a rational basis for the termination of pregnancy.

Next comes the question of the treatment of cases already in labour. Are we to proceed to extract, regardless of the shock caused,—a factor by no means to be neglected,—or to endeavour to make use of treatment by medical means alone? We should incline to a middle course, and do both. At the Bristol Royal Infirmary we have made a point of the following combination of methods when convulsions have commenced, and the patient is in labour: Venesection in all cases when there is any tendency to cyanosis, saline irrigation of the rectum, and saline injection into the subcutaneous tissue; the administration of morphia and of chloroform, if any manipulation is to be undertaken, and the hot, wet pack. Veratrum viride hypodermically certainly reduces the pulse-rate, but does not strike us as an essential method of treatment, or by itself one of much use, since we should consider the rapid pulse a symptom of the toxæmia. (A large mortality will at present invariably be met with in eclampsia no matter what treatment is made use of.) Labour we usually induce by manual dilatation of the cervix. Bozzi's dilator we have used and recommend, provided it is not made the means of producing full dilatation, since this, we think, is the point at which its use becomes risky. We complete dilatation by the hand after commencing with the dilator; in cases of rigid cervix it will be found a valuable instrument. We always hope for *post-partum* hemorrhage, but so far without success, although we have repeatedly endeavoured to cause it by the use of warm intra-uterine irrigation. In prophylaxis we induce labour if in pregnancy the excretion of urea remains below 200 grains per diem for three days in albuminuric cases.

It may be noted that all the drugs and methods employed are vaso-dilators, or lower pressure, *e.g.*, morphia in large doses, veratrum viride, and thyroid extract. Venesection and saline injection both lower blood-pressure.

We may sum up by saying: (1) Prophylaxis is best carried out by careful search for the symptoms of the pre-eclamptic stage, and appropriate treatment (purgation, restricted diet, diuretics and rest); (2) To deal with the convulsive stage, vaso-dilator drugs should be administered, and measures taken to lower blood-pressure; (3) Labour when begun should be terminated as rapidly as possible under anæsthesia; (4) If not begun should be induced if treatment has no effect, but that delay in waiting for the effect of treatment is risky unless the case is a mild one.

WALTER C. SWAYNE.

